# An Unexpected Cause of Diabetic Foot Ulcer Infection: Pasteurella multocida

**DOI:** 10.7759/cureus.103107

**Published:** 2026-02-06

**Authors:** Jesse H Wu, Paul M Shaniuk

**Affiliations:** 1 Medicine, Case Western Reserve University School of Medicine, Cleveland, USA; 2 Medicine, Louis Stokes Cleveland Department of Veterans Affairs Medical Center, Cleveland, USA

**Keywords:** amputation, diabetic foot complications, foot ulcer, osteomyelitis, pasteurella, pasteurella multocida, zoonotic infection

## Abstract

Diabetic foot infection (DFI) is a common complication of diabetes mellitus and a major cause of preventable morbidity and mortality in individuals with diabetes. Superficial DFI can lead to contiguous spread to soft tissue and bone as well as hematogenous spread and sepsis. It frequently involves typical pathogens such as *Staphylococcus aureus *and* Pseudomonas aeruginosa, *or it may be polymicrobial, involving Gram-negative bacilli and anaerobes. Atypical organisms can also be responsible, especially when environmental exposures are present. We report a rare case of *Pasteurella multocida* osteomyelitis in a 75-year-old man with diabetes with a chronic foot ulcer. The infection likely developed from indirect exposure to his household dog after a piece of dog food became trapped in his shoe, which went undetected due to peripheral neuropathy. After failing outpatient management, he presented with worsening foot swelling, erythema, and purulence and was admitted for intravenous antibiotics. His clinical status stabilized, and antibiotics were narrowed after the culture results, but ultimately, he was recommended to undergo a below-the-knee amputation. Amputation was done for definitive source control, and the patient had an uncomplicated post-surgical recovery. Although *Pasteurella multocida *is an uncommon cause for infection, it can lead to significant morbidity and mortality, and possible hematogenous spread and sepsis. This case study highlights the importance of thorough exposure history and culture-guided therapy in DFI, and it reviews aspects of *Pasteurella* infections that are associated with different outcomes.

## Introduction

Diabetic foot infection (DFI) is a serious complication of diabetes mellitus, often leading to significant morbidity and, in severe cases, lower extremity amputation. Around 19% to 24% of people worldwide with diabetes will develop a diabetic foot ulcer in their lifetime [1]. These infections typically arise due to a combination of peripheral neuropathy, peripheral arterial disease, and impaired immune response, which predisposes patients to ulcers that become secondarily infected. Osteomyelitis occurs in 15% of DFIs, of which 15% require amputation [2]. In the largest and longest evaluation of the risk of death among those with diabetes and lower extremity amputation, individuals with diabetes who have had lower extremity amputation were three times more likely to die than those who did not receive lower extremity amputation [3]. In addition, in any given year, >5% of those with diabetes and lower extremity amputation will die [3]. The most common pathogens involved include aerobic Gram-positive cocci (*Staphylococcus aureus*, *Streptococcus pyogenes*, and coagulase-negative staphylococci) and *Pseudomonas aeruginosa* [4]. Deep infections are more likely to be polymicrobial, involving Gram-negative bacilli and anaerobes. There is less information about the management of this common condition when the causative organism is atypical. We describe an uncommon pathogen as the cause of osteomyelitis in a diabetic foot ulcer.

## Case presentation

A 75-year-old male presented with a worsening left foot ulcer with redness, warmth, and swelling extending up the left calf. Past medical history was significant for type 2 diabetes mellitus (complicated by neuropathy), hypertension, benign prostatic hyperplasia, and dyslipidemia.

One week prior to admission, he presented to the podiatry outpatient clinic for a new left lateral heel wound along with a chronic plantar heel wound that was concerning for infection. He was started on amoxicillin-clavulanate. He reported using povidone-iodine-soaked dressings every day, which had resulted in soaking through to the bedsheets. One day prior to admission, he wrapped a plastic bag around his povidone-iodine-soaked dressing overnight, which caused the foot to become moist. He reported finding a flap of whitened, dead skin off the lateral heel in the morning, along with moist, swollen tissue on the foot. He trimmed the skin flap off and discarded it before going to his podiatry appointment on the day of admission.

He reported his wound started about six months ago when a piece of dog food got stuck in his shoe, and he could not feel it due to his neuropathy. It started as a blister and had worsened over time to approximately 1.5 cm x 3 cm, and he had been seeing a podiatrist for it. He had been adherent to amoxicillin-clavulanate for the week prior to admission, but he reported increasing fatigue. He reported left lower extremity redness and swelling, as well as loss of sensation in the bilateral feet and intermittent shock-like sensations running up his left leg. He denied fever, chills, weakness, or pain elsewhere. He was started on intravenous vancomycin and piperacillin-tazobactam in the emergency department and admitted to the hospital. A1C measured at the beginning of admission was 6.7%. Considering the lack of response to initial antibiotics and the history of a moist wound, empiric antibiotics aimed to target Gram-positive organisms, including methicillin-resistant *S. aureus*, as well as *P. aeruginosa* and anaerobes.

**Figure 1:**
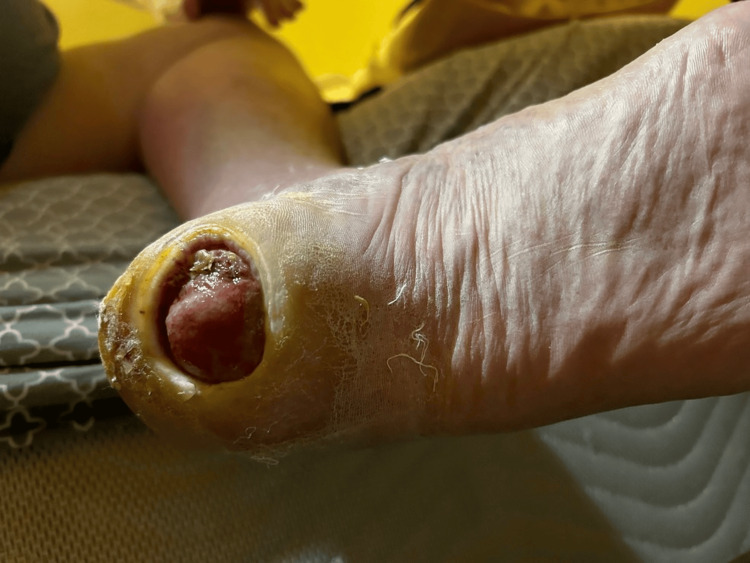
Necrotic plantar heel wound.

Vital signs at admission were 100.8°F, a heart rate of 90 beats per minute, a respiratory rate of 18 breaths per minute, and 98% oxygen saturation on room air. A skin exam demonstrated a 1.5 cm x 3 cm tense black necrotic ulcer on the lateral aspect of the left heel and a 2 cm x 2 cm ulcer on the plantar aspect of the left heel (Figure 1). A neurologic exam revealed loss of proprioception in the left hallux, with proprioception maintained in the right. There was a loss of crude touch near the toes bilaterally on the plantar surface. There was a palpable 2+ posterior tibial pulse on the right, but no palpable posterior tibial pulse on the left. There was a markedly dependent rubor in the left calf with warmth and pitting edema. A biphasic Doppler signal was found on the right, but it was not found on the left.

Labs demonstrated leukocytosis with neutrophilic predominance, elevated erythrocyte sedimentation rate (ESR), and elevated C-reactive protein (CRP), likely reflecting active infection. The remaining studies were otherwise unremarkable, including a blood culture that showed no growth after five days (Table 1).

**Table 1:** Lab studies. This patient's lab studies demonstrated elevated WBC, ESR, and CRP. CBC: complete blood count; WBC: white blood cell count; Hgb: hemoglobin; Hct: hematocrit; Plt: platelet count; Na: sodium; K: potassium; Cl: chloride; CO_2_: carbon dioxide or bicarbonate level; BUN: blood urea nitrogen; Cr: creatinine; ESR: erythrocyte sedimentation rate; CRP: C-reactive protein

Lab study	Parameter	Patient’s value	Reference range
CBC	WBC	11.5 k/cm^2^	4.5 - 11 k/cm^2^
	Hgb	13.2 g/dL	13.2 - 16.6 g/dL
	Hct	39.8%	38.3% - 48.6%
	Plt	323 k/cm^2^	150 - 400 k/cm^2^
Automated differential	Neutrophils	75.4%	40 - 60%
	Lymphocytes	12.9%	20 - 40%
	Monocytes	8.1%	4 - 8%
	Eosinophils	2.5%	1 - 3%
Procalcitonin	Procalcitonin	0.05 ng/mL	<0.1ng/mL
Serum chemistry	Na	140 mmol/L	135 - 145 mmol/L
	K	4.1 mmol/L	3.5 - 5 mmol/L
	Cl	106 mmol/L	96 - 106 mmol/L
	CO_2_	23 mmol/L	22 - 26 mmol/L
	BUN	12 mg/dL	7 - 20 mg/dL
	Cr	0.8 mg/dL	0.6 - 1.3mg/dL
	Glucose	130 mg/dL	70 - 100 mg/dL
	Albumin	3.8 g/dL	3.5 - 5.0 g/dL
	Total Protein	8.0 g/dL	6.0 - 8.3 g/dL
ESR	ESR	65 mm/hr	<20 mm/hr
CRP	CRP	7.36 mg/dL	<1.0mg/dL
Wound culture	N/A	*Pasteurella multocida* and diphtheroids	N/A
Antibiotic susceptibility testing	N/A	Does not produce beta-lactamase	N/A
Blood cultures	N/A	No growth after 5 days	N/A

**Figure 2:**
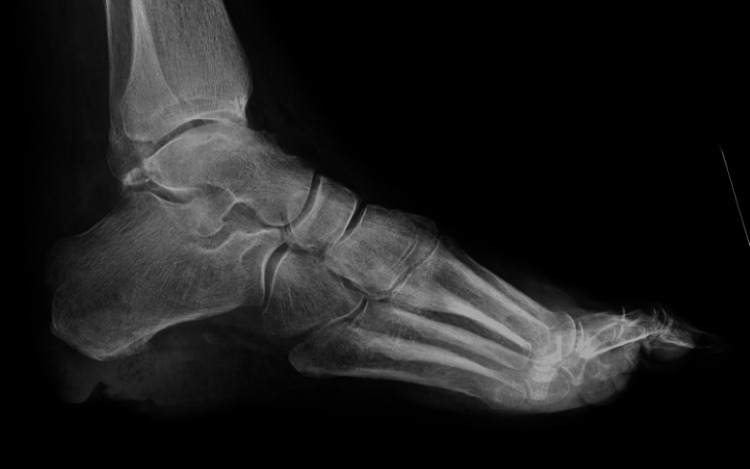
Plain film radiograph of the foot. This patient's X-ray image demonstrated sclerotic changes to the plantar calcaneus without signs of osteomyelitis.

**Figure 3:**
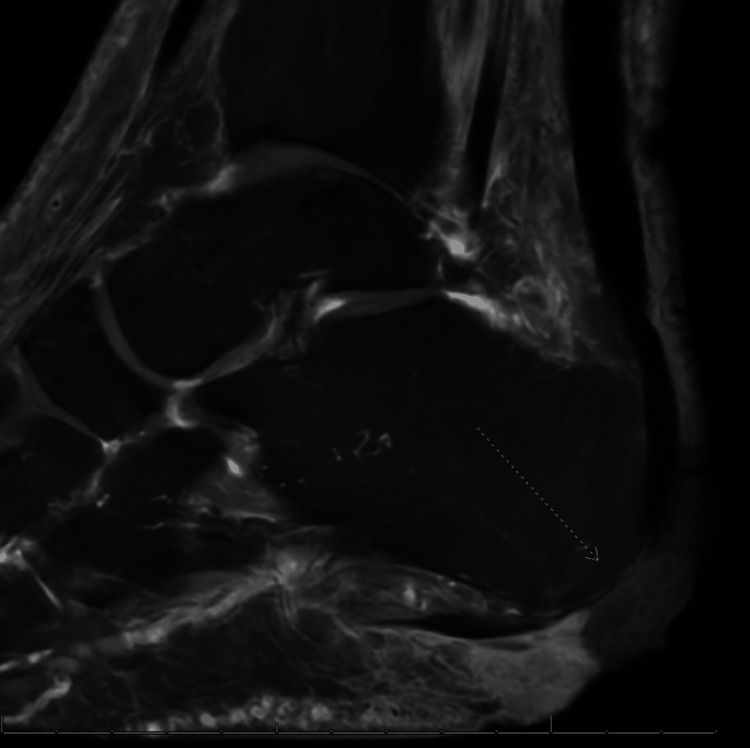
MRI of the calcaneus. This patient's MRI demonstrated osteomyelitis of the calcaneus (represented by the dotted arrow).

An X-ray of the foot demonstrated sclerotic changes to the plantar calcaneus without signs of osteomyelitis (Figure 2). Pulse volume recordings were obtained and were normal. MRI demonstrated osteomyelitis of the calcaneus (Figure 3).

His fatigue improved throughout the first day of admission on intravenous vancomycin and piperacillin-tazobactam. On the second day of admission, his wound was debrided by the Inpatient Podiatry service, yielding purulent fluid that was cultured. The bone probe at this time was positive, and the foot was deemed unsalvageable.

Wound cultures grew *Pasteurella multocida* and diphtheroids. Antibiotic susceptibility testing revealed that the *P. multocida* did not produce beta-lactamase. The history of the dog sniffing the wound and the dog food particle in his shoe was confirmed at this time, although he denied any history of the dog licking him or any other direct contact with the wound. His antibiotics were switched to intravenous ampicillin-sulbactam, as *P. multocida* grown was susceptible to beta-lactams, and the beta-lactamase inhibitor was added to cover the potential pathogenic role of the diphtheroids. Labs and clinical status remained stable throughout the hospital stay, and he received a below-the-knee amputation. He was transitioned to oral amoxicillin-clavulanate the day of amputation to complete a course of five days post-operation.

## Discussion

Foot infections are the most common cause of hospitalization in patients with diabetes, and they are a major cause of lower extremity amputations in the United States [5]. Risk factors for foot infection include sustaining a foot wound, recurrent or prolonged wounds (greater than 30 days), and peripheral artery disease [5]. Acute hematogenous osteomyelitis may be treated with antibiotics alone, but chronic osteomyelitis usually requires surgical management [6].

First discovered in 1878 by Edoardo Perroncito and isolated by Louis Pasteur in 1881 as the cause of fowl cholera, *Pasteurella multocida* is a Gram-negative coccobacillus that causes a variety of clinical syndromes. Multocida means “many killing,” referring to the fact that this bacterium is pathogenic for many species of animals [7]. It is a commensal bacterium in the oropharynx and upper respiratory tract of mammals and fowl [8]. Transmission to humans usually comes from cats and dogs, with reported carrier rates of 70% to 90% and 20% to 50%, respectively [9]. Based on our review of the literature, only a few cases exist in the past 15 years of *Pasteurella multocida* being a cause of diabetic skin and soft tissue infection or osteomyelitis of the foot [10-13].

Although *Pasteurella multocida* infection is most commonly associated with an exposure history, cases without an exposure history were more associated with bacteremia and increased hospital admissions. Exposure to household pets has been implicated in disease. However, infections not associated with an animal bite were also associated with increased severity of illness, with higher intensive care utilization and increased mortality [9]. The absence of bite or scratch in history may be associated with preexisting pulmonary disease, open wounds, or immunocompromise. Therefore, *Pasteurella multocida* in the absence of bite or scratch history should be considered an opportunistic infection [14].

In this patient, the foot wound began six months prior to presentation, but systemic symptoms (i.e., fatigue) were only present for the week prior to presentation. There is a possibility that this is a case of chronic calcaneal osteomyelitis, as it is impossible to truly know when the infection spread to the bone, but the time course is more consistent with acute osteomyelitis. Treatment failure with amoxicillin-clavulanate, which was started only one week prior to presentation, can likely be attributed to the onset of osteomyelitis necessitating surgical intervention prior to the start of antibiotic therapy.

Isolating a causative organism in osteomyelitis in a DFI can be challenging. Wound cultures are helpful in determining a causative organism regardless of bone involvement and are recommended by the Infectious Diseases Society of America (IDSA) guidelines on DFI [15]. The first step in the workup of suspected osteomyelitis is obtaining a plain film X-ray radiograph. Changes seen on X-ray may include periosteal reaction, regional osteopenia, cortical loss, or endosteal scalloping. Unfortunately, changes may take five to seven days in children and 10 to 14 days in adults to appear on a radiograph, making this a less sensitive test for osteomyelitis [16].

MRI is the mainstay in the diagnosis of osteomyelitis as the most sensitive and specific test [17]. Probe to bone test, which is where the provider probes the injury with a metal rod, scratching the innermost surface to feel for bone, has also shown a high diagnostic accuracy with a pooled sensitivity/specificity of 0.87 (95% confidence interval, 0.75-0.93) and 0.83 (95% CI, 0.65-0.93), respectively [18]. While the gold standard confirmatory test is the bone biopsy, this test may be a low-yield procedure, playing a minor role in diagnosis [19]. Often, results are concordant with clinical and radiologic impressions and rarely change management. A bone biopsy was not performed in this patient due to the likely low yield with an invasive procedure, considering antibiotic therapy had already been initiated a week prior to presentation. This does weaken the causative connection between *P. multocida* and osteomyelitis. But the pus obtained from wound debridement (which was cultured) was contiguous with the calcaneus, and along with the exposure history, a diagnosis of *P. multocida* osteomyelitis was made.

The pathogenic vs colonizing role of diphtheroids (i.e., non-diphtheriae *Corynebacterium* species, normal components of flora) is controversial, as reflected by the 2023 IDSA/International Working Group on the Diabetic Foot (IWGDF) Guidelines on the Diagnosis and Treatment of Diabetes-related Foot Infections [20]. The guidelines recommend having a careful sterile wound culture during debridement in order to avoid the growth of diphtheroids and other colonizers in the culture. In this case, the addition of a beta-lactamase inhibitor in the narrowed antibiotic regimen was to cover for the potential pathogenic role of diphtheroids in the osteomyelitis.

Prompt diagnosis of *P. multocida* infection or a new ulcer may improve outcomes in DFI, especially since many in this population lack sensation in the lower extremity due to neuropathy. Additional challenges in diabetes mellitus and osteomyelitis include poor wound healing and recurrence of infection. In this case, the patient could not feel the piece of dog food that was stuck in his shoe and only visually noticed it several days after a new wound had developed. The patient self-managed the wound at home prior to presenting for management, and foot wound debridement with culture of the drained purulent fluid occurred after hospitalization. The antibiotics were able to be narrowed to ampicillin-sulbactam and transitioned to oral amoxicillin-clavulanate after adequate source control with amputation. Amoxicillin-clavulanate was continued for five days post-operatively, as the most recent IDSA/IWGDF guidelines recommend a two to five-day course after surgical intervention [20].

The lack of a bone biopsy confirming the presence of* Pasteurella multocida* within the calcaneus is a limitation of this case report, as it weakens our conclusion of the causative link between *P. multocida* and the osteomyelitis diagnosed by MRI. However, due to the low likelihood of a result changing management of this patient and the costs associated, the bone biopsy was not performed, and the amputated foot was not sent for pathology. Post-operative recovery course was not complicated by any recurrence of infection, representing a response to the treatments given.

## Conclusions

Although *P. multocida* is an uncommon cause of osteomyelitis, it should be considered in patients at risk for opportunistic infections. Careful social history should include asking about a history of household pets or recent animal scratches or bites in a patient with a non-healing wound or deep-seated infection. Treatment depends on early clinical recognition and targeted antibiotic therapy, as well as surgical management for source control. While plain film radiography and MRI have substantial roles in diagnosis, antimicrobial therapy should also be guided by clinical impression and history. In this case, the 75-year-old male with an environmental history significant for dog food in the shoe and potential contact with dog saliva prompted clinical suspicion for *P. multocida* infection. Plain film radiography did not demonstrate evidence of osteomyelitis, but MRI did, demonstrating the value of MRI as a confirmatory test in cases of suspected osteomyelitis. There was a lack of specific animal bite or lick in his history, prompting a search for more disseminated infection within the bloodstream and further thought about the potential opportunistic nature of the infection. He received a below-the-knee amputation for definitive treatment and had no recurrence of infection after finishing a course of amoxicillin-clavulanate. Further research is needed to outline risk factors for localized versus disseminated infection as well as superficial versus deep tissue infection. The results of such studies may further guide the diagnosis and treatment of atypical pathogens in DFIs.
